# Genetic Profiling of *Aspergillus* Isolates with Varying Aflatoxin Production Potential from Different Maize-Growing Regions of Kenya

**DOI:** 10.3390/toxins11080467

**Published:** 2019-08-09

**Authors:** Richard Dooso Oloo, Sheila Okoth, Peter Wachira, Samuel Mutiga, Phillis Ochieng, Leah Kago, Fredrick Nganga, Jean-Baka Domelevo Entfellner, Sita Ghimire

**Affiliations:** 1 School of Biological Sciences, University of Nairobi, P.O. Box 30197-00100 Nairobi, Kenya; 2 Biosciences Eastern and Central Africa-International Livestock Research Institute (BecA-ILRI) Hub, P.O. Box 30709-00100 Nairobi, Kenya; 3 Department of Plant Pathology, University of Arkansas, Fayetteville, AR 72701, USA

**Keywords:** *Aspergillus flavus*, *Aspergillus minisclerotigenes*, internal transcribed spacer, calmodulin, aflatoxigenicity, aflatoxin biosynthesis genes, Kenya

## Abstract

Highly toxigenic strains of *Aspergillus flavus* have been reported to frequently contaminate maize, causing fatal aflatoxin poisoning in Kenya. To gain insights into the environmental and genetic factors that influence toxigenicity, fungi (*n* = 218) that were culturally identified as *A. flavus* were isolated from maize grains samples (*n* = 120) from three regions of Kenya. The fungi were further characterized to confirm their identities using a PCR-sequence analysis of the internal transcribed spacer (ITS) region of rDNA which also revealed all of them to be *A. flavus*. A subset of 72 isolates representing ITS sequence-based phylogeny cluster and the agroecological origin of maize samples was constituted for subsequent analysis. The analysis of partial calmodulin gene sequences showed that the subset consisted of *A. flavus* (87%) and *Aspergillus minisclerotigenes* (13%). No obvious association was detected between the presence of seven aflatoxin biosynthesis genes and fungal species or region. However, the presence of the *aflD* and *aflS* genes showed some association with aflatoxin production. The assessment of toxigenicity showed higher aflatoxin production potential in *A. minisclerotigenes* isolates. Given that *A. minisclerotigenes* were mainly observed in maize samples from Eastern Kenya, a known aflatoxin hotspot, we speculate that production of copious aflatoxin is an adaptative trait of this recently discovered species in the region.

## 1. Introduction

*Aspergillus flavus* is a filamentous and cosmopolitan fungus known for its ability to produce aflatoxins in food and feed. It attacks crops in the field, at harvest, and during transportation and storage. *Aspergillus flavus* is the main producer of aflatoxin B_1_ (AFB_1_), the second leading cause of aspergillosis that compromises immunity in humans [1]. Aflatoxin is a major food safety concern in Kenya as it frequently contaminates maize, a staple crop to approximately 90 percent of rural households [2]. Several incidences of fatal aflatoxicosis have been reported over the past three decades resulting from the consumption of aflatoxin-contaminated maize [3]. For example, the worst outbreak of aflatoxicosis in Eastern Kenya in 2004 resulted in 125 deaths [3,4,5,6,7]. It was hypothesized that the contamination occurred due to favorable weather conditions, an inappropriate harvesting time and the storage of maize in humid environments that favor *A. flavus* colonization [4]. A highly toxigenic strain of *Aspergillus,* known as *Aspergillus minisclerotigenes,* has been reported in Eastern Kenya [8], but its distribution in other maize-growing regions of Kenya is not known.

The sclerotia morphology are used to differentiate S and L strains of *A. flavus* [9]. The S-strain that produces considerable quantities of small dark sclerotia, is the most toxigenic as it produces high levels of B_1_ and B_2_ type aflatoxins. On the other hand, the L-strain produces yellow to bright green colonies with small amounts of sclerotia and inconsiderable amounts of B_1_ and B_2_ type aflatoxins [10]. The S-strains, originally identified as *A. flavus* were associated with human aflatoxicosis outbreak of 2004 in Kenya [5]. Later, they were identified to be distinct from *A. flavus* and closely related to *A. minisclerotigenes* [8]. *Aspergillus minisclerotigenes*, which was first described in 2008 [11], has typically small sclerotia but it is genetically different from other S-strain isolates [8]. Some of these isolates were previously described as *A. flavus* [12,13]. Isolates of *A. flavus* are known to produce only aflatoxin type B, whereas *A. minisclerotigenes* isolates produce both aflatoxins B and G [14]. The analysis of the aflatoxigenicity potential of *A. flavus* and *A. minisclerotigenes* isolates across maize-growing regions of Kenya would be necessary to develop an effective aflatoxin mitigation strategy for different maize production environments.

The characterization of *Aspergillus* section *Flavi* species based on cultural and morphological features is difficult due to interspecific similarities and intraspecific variability [15]. It is often not straightforward and demands more time [1]. In *A. flavus*, phenotypic disparity and genetic diversity have been reported [16]. Although the internal transcribed spacer (ITS) region of the ribosomal DNA sequence analysis has been used to identify fungi to species level, it may not differentiate between some closely related members of certain genera. Thus, genes such as β-tubulin, calmodulin and actin are used for accurate identification [17,18]. For example, recent studies in Portugal used calmodulin and ubiquitin gene markers to resolve genetic differences and to accurately identify *Aspergillus* species and other cryptic fungal species [19]. Thus, there is need to combine cultural, biochemical and robust molecular techniques, including the analysis of specific genes for the accurate identification and characterization of toxigenic fungal species.

Aflatoxins (AF) are derived from polyketides that are produced through a complex conversion pathway [20]. The aflatoxins production pathway consists of approximately 30 genes, some having ambiguous roles in aflatoxin biosynthesis [21]. The genes with clear roles in the pathway have been assigned *aflA* to *aflR*. The conversion of the primary metabolite, acetate, to the first stable AF biosynthesis intermediate, Norsolorinic Acid (NOR), is carried out by the action of *aflA*, *aflB*, and *aflC*. The *aflD* gene encodes an enzyme that catalyzes the conversion of NOR to Averantin. Averantin is converted through various steps to versicolorin A (VER A) through the action of *aflE* to *aflL* genes. The *aflM* converts VER A to demethylsterigmatocystin (DMST), which is converted to Sterigmatocystin (ST) by the action of *aflO*. The *aflP* converts ST and DMST to O methylsterigmatocystin (OMST) and dihydro-Omethylsterigmatocystin (DHOMST), respectively. The *aflQ* gene completes the pathway by converting OMST to aflatoxin B_1_ and G_1_ and DMDHST to aflatoxin B_2_ and G_2_ [22]. Two genes that are involved in the regulation of aflatoxin biosynthesis pathway are *aflR* and *aflS*. The *aflR* is a positive regulatory gene that activates the transcription of the structural genes of aflatoxin pathway [23]. The *aflS*, located next to the *aflR* gene in the aflatoxin gene cluster, is implicated in the regulation of transcription [24].

Aflatoxin biosynthetic pathway genes have been used to detect aflatoxin-producing fungi in various commodities [25]. The inability of *A. flavus* to produce aflatoxins is caused by differences in single nucleotide polymorphisms (SNPs), deletion of aflatoxin biosynthetic genes and mutations [26]. However, discrimination of aflatoxigenic strains and non-aflatoxigenic strains of *A. flavus* based on the presence or absence of aflatoxin biosynthetic pathway gene(s) has been difficult [27]. For example, a study by Okoth et al. [1] that analyzed the association between five aflatoxin biosynthesis genes and aflatoxigenicity in isolates from two maize-growing regions of Kenya could not establish a significant correlation. To expand the scope of the work by Okoth et al., [1], we used a wide panel of isolates from three regions of Kenya: Eastern Kenya, a known aflatoxin hotspot; Western Kenya, the grain basket of the country, and the Coast, with some significant maize farming and no major reports of aflatoxin contamination [3,28], to assess the relationship between toxigenicity and the presence of the AF genes. The objectives of this study were; i) to characterize isolates and to develop molecular tools for the differentiation of *A. flavus* and *A. minisclerotigenes*, ii) to compare the aflatoxin production potential (aflatoxigenicity) between isolates of *A. flavus* and *A. minisclerotigenes* from different maize-growing regions of Kenya, and iii) to assess the relationship between seven aflatoxin biosynthesis genes and total aflatoxin production by *Aspergillus* isolates. This study detected both *A. flavus* and *A. minisclerotigenes* in Kenya and isolates of *A. minisclerotigenes* were more toxigenic than *A. flavus* isolates. It was also noted that the aflatoxin production profile was dependent on the geographical origin of the isolate. Furthermore, a positive correlation was observed between two aflatoxin genes (*aflD* and *aflS*) and total aflatoxins production.

## 2. Results

### 2.1. Cultural Characterization of Aspergillus Isolates

*Aspergillus* section *Flavi* isolates (*n* = 258) were isolated from the maize samples (*n* = 120) collected from the three regions of Kenya. These isolates had yellow to green colonies with white mycelia at the edges and cream reverse on potato dextrose agar (PDA). Based on an orange appearance of the colonies in the reverse of a Petri dish containing the selective Aspergillus Flavus Parasiticus Agar (AFPA) medium, the majority (218/258, 84%) of the isolates were putatively identified as *A. flavus*. Owing to the scope of this study, isolates that were not culturally identified as *A. flavus* were dropped from the subsequent analyses. The distribution of the putative *A. flavus* isolates, in increasing order, across the three major regions of Kenya were as follows: Eastern (35/218, 16%), Coast (48/218, 22%) and Western (135/218, 62%).

### 2.2. Characterization of Aspergillus Isolates Based on Sequences of the ITS Region

**Figure 1:**
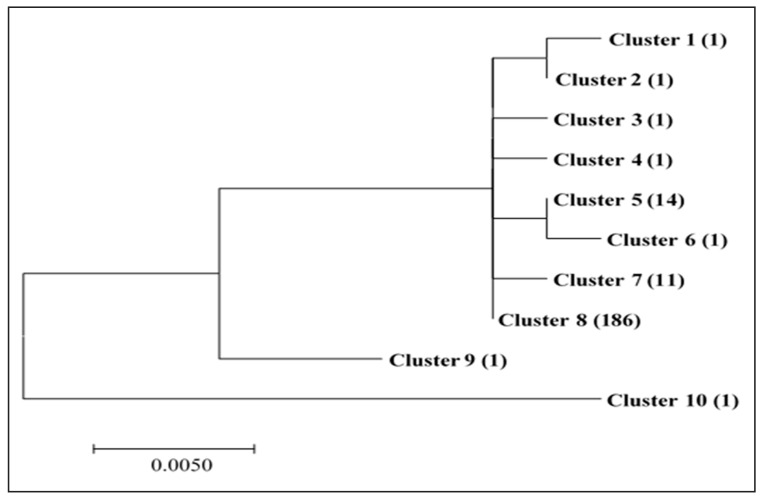
Genetic relationship of isolates of *Aspergillus flavus* from three regions of Kenya, as characterized using sequences of the internal transcribed spacer (ITS) region. The dendrogram was derived using the Tamura 3-parameter model in MEGA 7 [29].

Isolates that were identified as *A. flavus* (*n* = 218) by a culture-based method were sequenced for the ITS region and the sequences were deposited to the National Center for Biotechnology Information (NCBI) with accession numbers ranging from MK493797 to MK494014. When the sequences of these isolates were compared with those that are available in NCBI database using Basic Local Alignment Tool (BLASTn), they were found to have a high homology (≥99%) to *A. flavus*. Phylogenic analyses conducted in MEGA 7 using Maximum Likelihood based on the Tamura 3-parameter model [29] grouped 218 isolates into ten genetically distinct clusters, (Figure 1). Based on genetic distances, Cluster 10 was more divergent from the rest. Most isolates belonged to Cluster 8 (*n* = 186, 85%), followed by Clusters 5 (*n* = 14, 6%) and then Cluster 7 (*n* = 11, 5%). The remaining other seven Clusters had one isolate each (Table S1).

Out of 593 nucleotides (starting from the sequence; TTAAGTTCAGCGGGTATCCC) used in the analyses of ITS region, only 30 nucleotide positions exhibited polymorphism. There was only one insertion/deletion observed (in Cluster 3), and the rest were substitutions. One isolate in Cluster 10 (Isolate 135) exhibited the highest percentage (43%, *n* = 13) of the observed polymorphisms. Another isolate in Cluster 9 (Isolate 206) showed three unique polymorphisms and shared five SNPs with Isolate 135. The remaining 216 isolates in the other eight clusters showed different polymorphisms in only nine nucleotide positions (Table S2). 

### 2.3. Identification and Analysis of Polymorphism in Calmodulin Gene Sequences 

**Figure 2:**
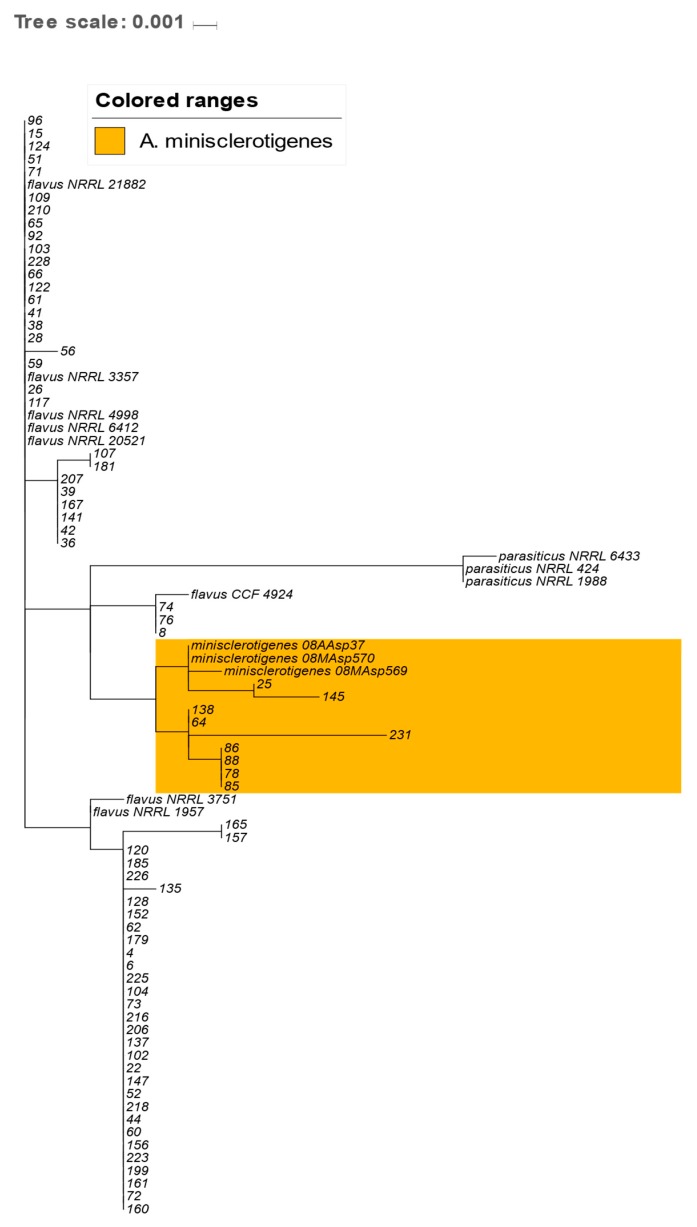
Genetic relationship of *Aspergillus flavus* and *Aspergillus minisclerotigenes* isolates based on partial calmodulin gene sequences. Phylogenetic tree drawn to scale with reference sequences of respective species obtained from National Center for Biotechnology Information (NCBI) Database. Orange color represents the *Aspergillus minisclerotigenes* clade.

The subset (*n* = 72) of the isolates chosen to represent the distinct genetic clusters (based on ITS region data) and the diverse regions were further sequenced for a partial calmodulin gene. The sequences were deposited at the NCBI with accession numbers MK494015 to MK494086. When the sample sequences of the partial calmodulin gene were compared with sequences at NCBI, the isolates were reclassified into *A. flavus* (87.5%, n = 63) and *A. minisclerotigenes* (12.5%, *n* = 9). Thirteen distinct sequences were obtained from calmodulin gene analysis when duplicate sequences were removed. A phylogenetic analysis of the calmodulin gene sequences showed two major genetic clades, each for *A. flavus* and *A. minisclerotigenes*. Unlike *A. minisclerotigenes*, several subclades were observed in the clade of *A. flavus*. Regarding regional distribution of the isolates identified by calmodulin sequencing, *A. flavus* occurred throughout the collection sites. Of the nine *A. minisclerotigenes* isolates, seven originated from Eastern Kenya, and one isolate each from Western and Coastal regions (Figure 2).

Polymorphism was observed at thirty nucleotide positions within the 720-bp alignment of the calmodulin gene (starting from the sequence; CACCATTTTTACAGCCGCAA). Of the observed polymorphisms, the majority were nucleotide substitutions, with only three (10%) being insertion/deletions. Generally, *A. minisclerotigenes* had more variants (SNPs) than *A. flavus* within the region, (Table S3). Overall, there were six SNPs that could potentially differentiate between *A. minisclerotigenes* from *A. flavus*. Based on these sequences, only two SNPs at positions 424 and 701 within the partial calmodulin gene, could discriminate clearly between the two species (Table 1).

### 2.4. Analysis of the Genetic Relationships between A. flavus and A. minisclerotigenes Based on Concatenated Sequences of ITS Region and Partial Calmodulin Gene

**Figure 3:**
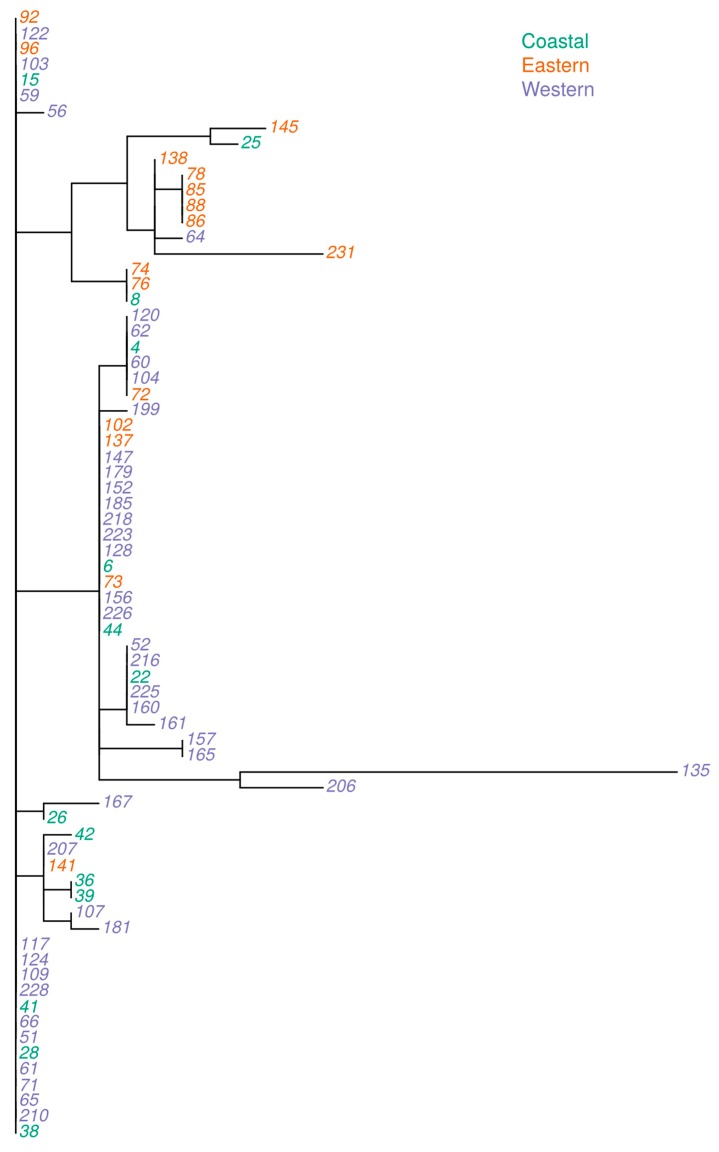
Genetic relationship among isolates of *Aspergillus flavus* and *Aspergillus minisclerotigenes* based on concatenated sequences of internal transcribed spacer (ITS) and partial calmodulin gene with the geographical origin of the isolate color coded (Eastern: Machakos, Makueni and Meru; Western: Bungoma, Kitale Nandi and Rachuonyo; Coast: Kilifi).

When the ITS region and partial calmodulin sequences were aligned and merged, the resultant concatenated sequence was 1313 nucleotides. Phylogenetic analyses of concatenated sequences for 72 isolates grouped them into 27 genetic clades (herein after named alleles). Based on this analysis, *A. minisclerotigenes* formed a unique clade together with three *A. flavus* isolates. Two of the isolates that grouped together with *A. minisclerotigenes* were from Eastern Kenya. The clade with *A. minisclerotigenes* had 78% (7 out of the 9) isolates that originated from Eastern region. The remaining clades had only *A. flavus* isolates showing more variability and were distributed across all three regions (Figure 3).

**Figure 4:**
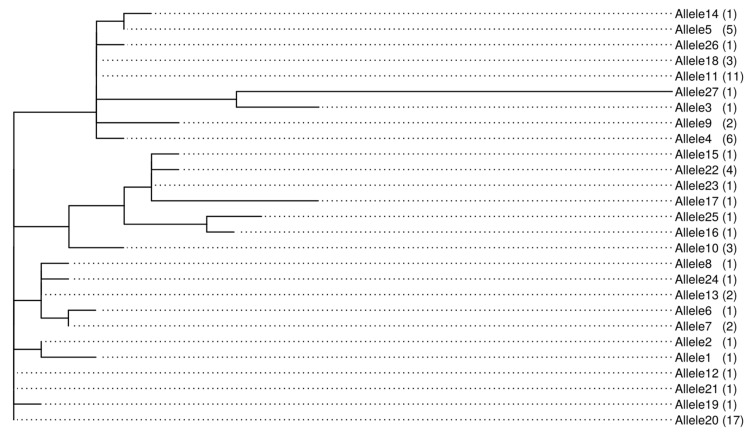
Allelic relationship unique sequences derived from concatenated ITS and partial calmodulin gene sequences. Maximum-likelihood phylogenetic tree generated using the Randomized Axelerated Maximum Likelihood (RAxML) software with a Generalized Time-Reversible nucleotide substitution model with classes of evolutionary rate determined according to a discrete Gamma law (GTRGAMMA model). The total count of sequences bearing the allele is indicated in brackets.

The distinct alleles (named in a range from 1 to 27) derived from the concatenated sequences were called and used for further phylogenetic analyses (Figure 4). Most of the isolates that had clustered together in individual ITS and calmodulin gene phylogenetic analyses were separated using the combined sequences. Genetic distances varied for the isolates, with Allele 27 having highest genetic divergence. Alleles 12, 20 and 21 contained isolates which were more similar than the rest of the groups (Figure 4). Allelic group 12 and 21 had one isolate each from the Western region whereas allelic group 20 had 17 isolates which originated from across the three regions, with the highest proportion (59%, *n* = 10 isolates) from the Western region. Moreover, all isolates in these three allelic groups were confirmed to be *A. flavus* based on ITS and calmodulin markers.

### 2.5. Aflatoxin Production in Culture Media

When the subset of isolates (*n* = 72) were grown on Yeast Extract Sucrose Agar (YESA), they produced aflatoxin at varying potentials. Aflatoxin production potential ranged from 2 to 118,800 µg kg^−1^, with a mean of 8386 µg kg^−1^ and a high standard deviation of 21,838 µg kg^−1^. The spread of aflatoxin production potential differed in isolates from different regions, although a Kruskal–Wallis test of significance yielded a high *p*-value (*p* = 0.26). The Eastern region had the highest standard deviation (26,310 µg kg^−1^, *n* = 16) followed by the Coastal (21,557 µg kg^−1^, *n* = 14) and then Western (19,212 µg kg^−1^, *n* = 42) regions. An arbitrary aflatoxin production potential (APP) limit of 100 µg kg^−1^ was applied in this study to distinguish highly toxigenic and low toxigenic isolates [30]. Out of 72 isolates tested, 28 isolates (39%) were highly toxigenic (APP over 100 µg kg^−1^). The increasing order of the percentages of the highly toxigenicity isolates by region are as follows: Western (50%), Eastern (29%) and Coast (21%). Approximately all low toxigenic strains produced mainly aflatoxin G_1_ (Figure S1). Sixty-one percent of highly toxigenic strains (*n* = 17) produced mainly AFBs, 25% (*n* = 7) produced only AFGs and the rest produced both. Ten out of thirteen isolates with APP above 10,000 µg kg^−1^ , produced mostly AFB_1_ (Figure S2).

When toxigenic potential was compared between species, a Wilcoxon rank sum test found a significantly (*p* < 0.001) higher aflatoxin production potential in *A. minisclerotigenes* (mean = 45,744 µgkg^−1^, median = 41,884 µgkg^−1^, *n* = 9) compared to *A. flavus* (mean, 3049 µg kg^−1^, median, 54 µg kg^−1^, *n* = 63). Unlike *A. flavus* isolates which had APPs that varied from low to high, all *A. minisclerotigenes* had a high APP (Figure S3). Interestingly, *A. minisclerotigenes* from Eastern Kenya produced higher aflatoxin Bs (range: 10,404 to 81,838 µgkg^−1^, median = 41,831 µg kg^−1^, mean = 42,895 µg kg^−1^, SD = 22,820 µg kg^−1^) compared to those from Western Kenya (*n* = 1, value = 4159 µg kg^−1^) and Coastal (*n* = 1, value = 9137 µg kg^−1^) regions produced more aflatoxin Gs. Conversely, *A. flavus* from Eastern Kenya produced the lowest levels of aflatoxin Bs: out of 24 *A. flavus* with a toxigenic potential over 2 µg kg^−1^, only one isolate was from the Eastern region. Furthermore, *A. minisclerotigenes* from Eastern Kenya produced significantly (*p* < 0.005) lower aflatoxin Gs compared to those from Western and Coastal regions. 

A significantly (*p* < 0.001) higher positive correlation was observed between the production of aflatoxin B_1_ and B_2_ (*r* = 0.96) than aflatoxin G_1_ and G_2_ (*r* = 0.92). Similarly, the correlation (*r* = 0.83) between aflatoxin B_1_ and total aflatoxin (*p* < 0.001) was higher than that of G_1_ and total aflatoxin (*r* = 0.727, *p* < 0.001).

### 2.6. Relationship between Aflatoxin Production in Culture Media and the Natural Contamination of Maize

The natural contamination in maize samples (*n* = 56) from which the *Aspergillus* isolates had been isolated was compared with the corresponding aflatoxin production potential of the isolates (*n* = 72) in culture media. The aflatoxin contamination of the maize samples ranged from 4 to 10,505 µg kg^−1^. To test for the relationship between the two parameters, means of the aflatoxin potential of the isolates for isolates that were obtained from an individual maize sample were computed and compared with the natural contamination level. When the total aflatoxin in the naturally contaminated maize was compared with the total aflatoxin production potential, a marginally significant positive correlation (*r* = 0.02, *p* = 0.046) was observed. A similar marginal relationship was observed when APP based on AFB_1_ was compared with the natural contamination of maize with AFB_1_. However, there was a lack of relationship (*p* > 0.05) between natural contamination and APP among other aflatoxin types.

### 2.7. Aflatoxin Biosynthesis Gene Profile and Its Relationship with Aflatoxin Production Potential

Seventy-two isolates were screened for seven aflatoxin biosynthesis genes using the gel-electrophoresis method. The *aflD* and *aflQ* that were each amplified using two different primer sets; *aflD*’ (AflD-1/AflD-2rev primers), *aflD”* (Nor1-F/Nor-R primers), *aflQ’* (AflQ-1/AflQ-2rev primers) and *aflQ”* (Ord1-gF/ Ord1-gR primers) gave different results.

Overall, the frequencies of the alleles of the aflatoxin biosynthesis genes were as follows: *aflD*’ (71%), *aflD*” (83%), *aflM* (89%), *aflO* (94%), *aflP* (96%), *aflQ*’ (88%), *aflQ*” (100%), *aflR* (81%) and *aflS* (78%). Among the regions, the percentage abundance of the genes were: Coastal (64%, 79%, 79%, 93%, 93%, 93%, 100%, 64% and 64%), Eastern (75%, 94%, 100%, 100%, 100%, 94%, 100%, 94% and 88%) and Western (71%, 81%, 88%, 93%, 95%, 83%, 100%, 81% and 79%) in the order; *aflD’*, *aflD”*, *aflM*, *aflO*, *aflP*, *aflQ’*, *aflQ”*, *aflR* and *aflS*, respectively. Within the species, the frequency of the alleles was as follows; *A. flavus* (73%, 81%, 89%, 94%, 95%, 86%, 100%, 83% and 76%,) and *A. minisclerotigenes* (56%, 100%, 89%, 100%, 100%, 100%, 100%, 67% and 89%,) in the order; *aflD*’, *aflD*”, *aflM*, *aflO*, *aflP*, *aflQ*’, *aflQ*”, *aflR* and *aflS*, respectively. When the frequency of the genes was compared between the species, it was generally observed that *A. minisclerotigenes* had more of the tested genes present than *A. flavus*.

The association between aflatoxin production potential and the presence of individual and/or a combination of them (or the risk factor analysis) was assessed using a stepwise regression model. To test different models, aflatoxin data were log-transformed. The best model which explained the aflatoxin production potential of the isolates was identified using the Akaike information criterion (AIC) (Table 2). Based on this analysis, the genes which significantly associated with aflatoxin production potential were *aflD*” and *aflS* (*p* < 0.05). They were significant predictors, the presence of which increased total aflatoxin production levels by at least 177 and 41 µg kg^−1^, respectively. The combined effect of the two genes would increase aflatoxin production potential by approximately 140 µg kg^−1^. Isolates with both (interaction) *aflD*” and *aflR* would reduce aflatoxin production potential by approximately 1 µg kg^−1^ (*p* = 0.028). The other genes did not show a significant influence on the toxigenicity of the isolates. Although the interaction between *aflD*” and *aflM* contributed to an improvement of the stepwise regression model, it did not significantly affect aflatoxigenicity potential.

**Figure 5:**
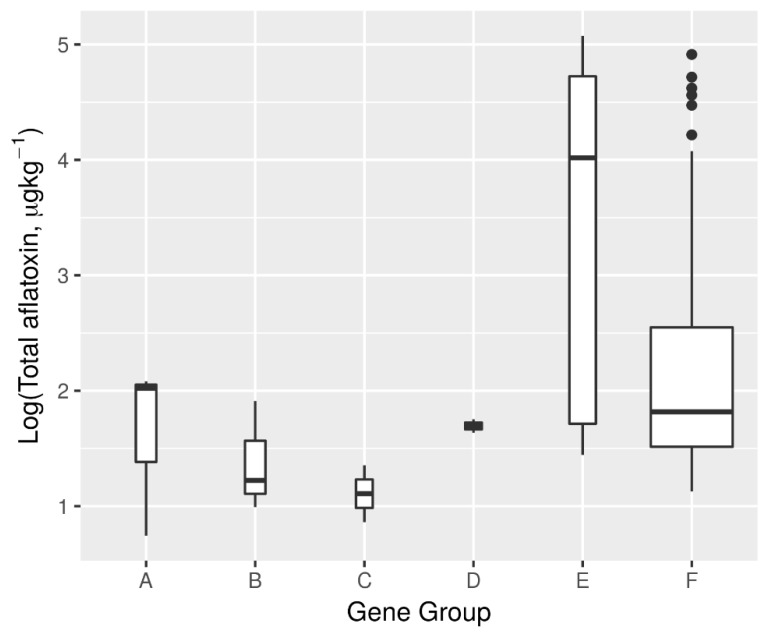
Effect of aflatoxin biosynthesis gene groups on the production of total aflatoxin by *Aspergillus flavus and Aspergillus minisclerotigenes*. Gene combinations (A, 000000001, *n* = 3; B, 000110011, *n* = 3; C, 001110001, *n* = 2; D, 001111011, *n* = 2; E, 011111111, *n* = 5; and F, 111111111, *n* = 48). Genes ordered as follows: *aflD* (aflD-1/aflD-2rev primers), *aflD* (Nor1-F and Nor-R primers), *aflM*, *aflO*, *aflP*, *aflQ* (AflQ-1/AflQ-2rev primers), *aflQ* (Ord1-gF/ Ord1-gR primers), *aflR* and *aflS*). Appearance of codes 1 or 0 denotes the gene that is present or absent, respectively in the concatenation.

Encoding gene presence (1) and absence (0) and then concatenating the resultant binary bits (in the order: *aflD* (aflD-1/aflD-2rev primers), *aflD* (Nor1-F and Nor-R primers), *aflM*, *aflO*, *aflP*, *aflQ* (AflQ-1/AflQ-2rev primers), *aflQ* (Ord1-gF/ Ord1-gR primers), *aflR* and *aflS*), to have one binary vector for each isolate, 16 gene combinations were obtained. Ten combinations had one *Aspergillus* isolate each, while the remaining six combinations, which were analyzed further, had more than one isolate. The analysis of median aflatoxin production by the isolates of the respective gene effect groups showed that isolates with the gene combination of group E had the highest capacity of aflatoxin production (Median = 10,470.3 µg kg^−1^). Isolates in group C had the lowest aflatoxin production potential with a median of 11.9 µg kg^−1^ (Figure 5).

### 2.8. Genetic Relatedness and Aflatoxin Production Potential of Isolates

**Figure 6:**
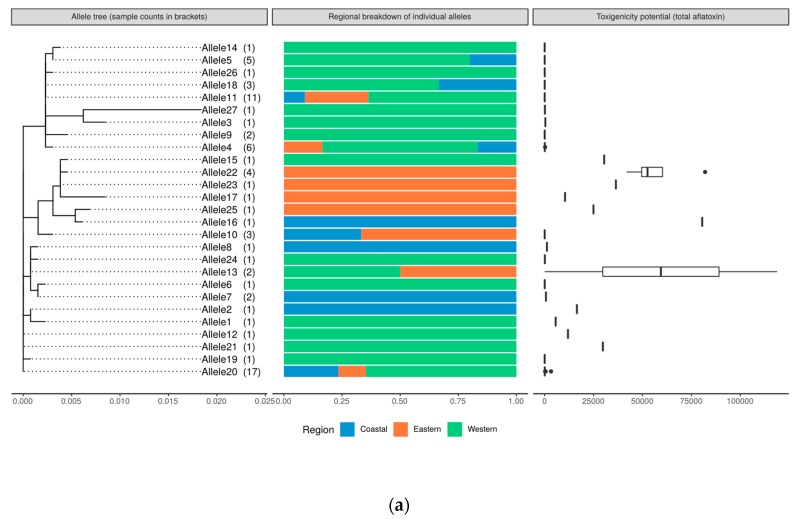

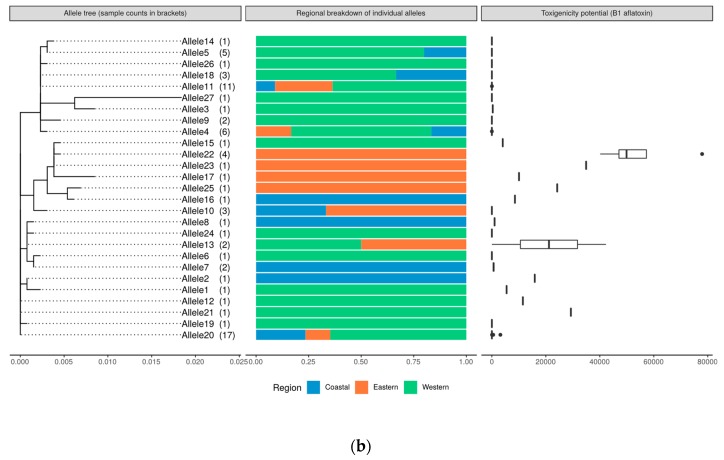
(**a**) Genetic relatedness, geographical location and aflatoxin B_1_ production potential in *Aspergillus flavus* and *Aspergillus minisclerotigenes* isolates from three regions of Kenya. Colors denote sampling regions. Phylogenetic tree generated using RAxML, GTRGAMMA model. (**b**) Genetic relatedness, geographical location and total Aflatoxin production potential in *Aspergillus flavus* and *Aspergillus minisclerotigenes* isolates from three regions of Kenya. Colors denote sampling regions. Phylogenetic tree generated using RAxML, GTRGAMMA model.

The similarities of the isolates were assessed based on allelic diversity, inferred by counting the number of distinct alleles represented in each region. The diversity of the isolates was highest in the Western region followed by the Coastal region and lowest in the Eastern region, with 18, 10 and 9 out of 27 distinct alleles, respectively (Figure S4). Aflatoxin production potentials (Total AF and AFB_1_) were compared among the distinct allelic clusters and geographical regions (Figure 6a,b). Fungal isolates were well classified into two species based on calmodulin sequence data. Alleles 15, 16, 17, 22, 23 and 25 consisted of isolates making the clade of *A. minisclerotigenes*, with most of them originating from Eastern Kenya. This group had highly toxigenic isolates only (Total AF 10,432–81,982 µg kg^−1^; AFB_1_, 4038–77,909 µg kg^−1^). Allele 13 had two isolates of contrasting APP capacities. Allele 11 consisted of eleven isolates of low APP (ranges: Total AF, 14–68 µg kg^−1^; AFB_1_, 0–27 µg kg^−1^).

## 3. Discussion

Enriching the knowledge on the genetics and spread of toxigenic strains of *Aspergillus* in Kenya was the focus of this study. It strengthens previous studies by expanding the geospatial scope and the sample size using isolates of *Aspergillus* that were cultured from three maize-growing regions of Kenya. We sought to establish whether the aflatoxin biosynthetic genes and diversity, based on the ITS region and calmodulin gene, are associated with aflatoxin production potential in isolates of *A. flavus* and *A. minisclerotigenes*. We tested whether the two genetic regions could discriminate between the two species of *Aspergillus* and whether the selected aflatoxin biosynthetic gene clusters were distinct among the fungal collections. The findings show the utility of specific genomic regions (single nucleotide polymorphisms) in differentiating between *A. flavus* and *A. minisclerotigenes* and a confirmation of the occurrence of the most toxigenic species in an aflatoxin hotspot. This proves significant to pathologists and plant breeders focusing on formulation of aflatoxin mitigation measures. In this study, we attempted to establish the relationships between aflatoxin production potential in the culture medium and the natural contamination of maize. However, this relationship could have been affected by other factors (e.g., temperature, rainfall, humidity, storage conditions, other microbial species) beyond the scope of this study.

The use of cultural and molecular methods is common for the authentic identification of fungal species [15]. We used AFPA for the selective isolation of putative *A. flavus* followed by the PCR amplification of ITS and calmodulin genes. We observed that the cultural method and the ITS marker were not enough for differentiating between *A. flavus* and *A. minisclerotigenes* isolates. The calmodulin gene marker separated the isolates identified as *A. flavus* using the ITS marker into *A. flavus* and a few *A. minisclerotigenes*. This study identified SNPs within the calmodulin gene which could be used to discriminate between *A. flavus* and *A. minisclerotigenes*. All *A. minisclerotigenes* isolates except two were from Eastern Kenya. *Aspergillus flavus* was common across all the three regions. These findings indicate that *A. flavus* and *A. minisclerotigenes* are spatially distributed. This observation is in agreement with previous studies and confirms the geographical effects on the evolution of the two species [1]. Phylogenetic analyses of the combined ITS and calmodulin sequences showed wider variation than the two markers analyzed separately, signifying importance of the integration of more than one molecular marker in characterization.

Aflatoxin production potential differed between *A. flavus* and *A. minisclerotigenes*. Isolates tested in this study produced aflatoxins in between 2.5 and 118,800 µg kg^−1^. Previous studies on *A. flavus* isolates from Kenya and Bangladesh reported a toxigenic potential of up to 152,966 µg kg^−1^ [27,32]. High variation among isolates for aflatoxin production potential in the current study could be related to genetic recombination among toxigenic and atoxigenic strains [33]. Isolates clustered together on ITS and calmodulin sequences phylogeny had highly variable aflatoxin production ability. For instance, the two isolates which the recorded highest and lowest aflatoxin production ability, respectively, were from the same allelic group. Several studies have found that the mycotoxin producing ability of putative *A. flavus* is variable and not only dependent on strain but also affected by substrate types [34] and the geographical origin of the isolates [35,36,37]. This study also revealed the presence of high aflatoxigenic strains in all three regions, which could be attributed to high genetic variability, aflatoxin heritability, and recombination in the aflatoxin gene cluster [38]. It has been previously reported that not every *A. flavus* strain has the capacity to produce aflatoxins, and those that do normally produce aflatoxins B_1_ and B_2_ only [14]. However, some *A. flavus* isolates in this study produced both AFBs and AFGs and some produced only AFGs but at low levels. Isolates of *A. minisclerotigenes* are reported to produce both AFBs and AFGs [11], but this study found one *A. minisclerotigenes* isolate that produced only AFBs. Similar results were reported by Okoth et al., [1]. *Aspergillus minisclerotigenes* had higher toxigenic potential than *A. flavus*. All *A. minisclerotigenes* were highly toxigenic and most of them were isolated from Eastern Kenya. 

We observed a higher toxigenicity potential and high frequency of *A. minisclerotigenes* in Eastern Kenya, a region with frequent cases of aflatoxicosis [5]. The sub-humid and semi-arid agroecological zones, which are predominant in Eastern Kenya, have been reported to have high levels of aflatoxin contamination in maize [3]. Furthermore, the region has been reported to harbor deadly strains of *A. flavus* [8] and AFB_1_, the most toxic and carcinogenic, is the main aflatoxin type reported [32]. It is plausible to conclude that the high level of maize contamination in Eastern Kenya is caused by the presence of highly toxigenic strains of *A. minisclerotigenes*. Indeed all *A. minisclerotigenes* isolates produced copious amount of AFBs while those from Western and Coastal Kenya produced more AFGs. The diversity of the *Aspergillus* strains was lowest in the Eastern region but had highest average toxigenic potential. It is possible that *A. minisclerotigenes* evolved from *A. flavus* in an endeavor to cope with harsh drought conditions in Eastern Kenya [39].

This study found a higher correlation between the production of total aflatoxins and AFBs than total aflatoxin and AFGs. The majority of isolates analyzed in study were *A. flavus*, a species that is mainly known to produce AFBs [9,14,32]. It is presumed that aflatoxin contamination in maize is influenced by the toxigenicity potential of colonizing isolates. However, in the current study, a marginal association was observed between the natural contamination of maize and the toxigenicity potential of the isolates possibly due to sampling artifact. For example, different sub-sets of a maize sample were used in fungal isolation and quantification of natural contamination. Furthermore, competition between other resident microbes and toxigenic *Aspergillus* strains for substrates could have also influenced the observations [40]. The variation in the physical and chemical composition of kernels, factors associated with maize genotype and environment, could also affect the growth and toxin production in toxigenic *Aspergillus* strains [41]. 

We observed a marginal correlation between the frequency of aflatoxin biosynthesis genes and aflatoxin production potential. Since all the isolates in this study produced aflatoxins, we anticipated PCR amplification for all seven aflatoxin biosynthesis genes. However, the amplification of some genes failed in some isolates. Some isolates had a successful amplification for all seven genes but had low aflatoxin production. This marginal association could be attributed to intraspecific and interspecific genetic mutations within the targeted binding site of the primers, defects at various protein and molecular levels [11,42,43], and a low copy number of target genes. Our findings have clearly indicated that the presence of the seven tested aflatoxin biosynthesis genes in the two *Aspergillus* species is not enough to differentiate and characterize the aflatoxigenic strains, but the presence of certain genes had a positive effect on aflatoxin production.

## 4. Conclusions

This research emphasizes the importance of the integration of morphological and molecular methods for the identification and characterization of *Aspergillus* spp. A partial calmodulin gene marker proved important in discriminating between *A. flavus* and *A. minisclerotigenes*. A spatial distribution of the toxigenic *A. minisclerotigenes* that mainly produce AFBs was evident in Eastern Kenya, where high aflatoxin contamination of maize and fatal aflatoxicosis have been reported. *Aspergillus flavus* were more common across all the regions and varied in the production of aflatoxins. We observed a marginal correlation between the frequency of seven aflatoxin biosynthesis genes and aflatoxin production potential. However, the presence of *aflD* and *aflS* significantly increased the amount of total aflatoxin production. To gain a better understanding of the genetics for aflatoxin production in *Aspergillus* spp., there is a need to conduct a study involving a larger number of isolates collected from a wider geographical location in Kenya with varying toxin production potential against high resolution genetic markers.

## 5. Materials and Methods 

### 5.1. Study Sites and Survey Design

Participants who provided maize samples for use in this study were households from eight sites, representing three regions of Kenya namely; Western Kenya (Rachuonyo, Bungoma North, Kitale, Nandi), Eastern Kenya (Machakos, Meru, Makueni) and Coastal Kenya (Kilifi) (Figure S5). Western Kenya produces more than three-quarters of the total maize consumed in the country and has no report of serious aflatoxicosis outbreaks [32]. High levels of maize contamination were reported in survey that was conducted in 2010 within the drought-prone parts of Rachuonyo. Eastern Kenya is prone to drought and is a known hotspot for aflatoxin contamination and deadly aflatoxicosis [3]. Coastal Kenya has some significant maize production activities and has not had any reports of major contamination or aflatoxicosis. A cross-sectional survey design was used to purposively sample maize grains from households who met the following conditions: they kept cattle, had a child of below five years and a breast-feeding mother. These sampling criteria were set by another ongoing study named Safe Food Safe Diary [44], which our efforts were to complement. Households meeting the sampling criteria were pooled and randomly picked to obtain the final sample size, based on a formula by Cochran [45]. A total of 15 households were selected to provide the samples of maize that had been harvested in year 2017.

### 5.2. Sample Collection

Sampling was conducted between July and August 2017. Approximately 2 kg of shelled maize grain were collected from the storage sheds of each household. Samples were scooped from either 90-kg polystyrene bags using a double-tube spear or from a 10-kg polystyrene bag using a closed spear, as was described in an earlier study [46]. The sampling devices was inserted through the upper open point of the polystyrene bag to draw grain at different depths [28]. The majority of the households had less than ten 90-kg bags. For the few households with more than ten 90-kg bags, ten bags were randomly selected for sampling [32]. Grain was packaged into paper envelopes and sealed prior to transportation for laboratory analysis. Samples were kept in a cold room at 4 °C until the time of analysis at Biosciences eastern and central Africa - International Livestock Research Institute (BecA-ILRI) Hub, Nairobi, Kenya.

### 5.3. Isolation and Identification of Aspergillus flavus

Maize kernels (*n* = 12) from each sample were assessed for internal colonization by fungi using the direct plating technique [47]. Four kernels were surface sterilized by dipping in diluted commercial bleach (2.5% sodium hypochlorite) for 1 min prior to rinsing thrice in sterile distilled water. Kernels were plated in three replicates on ¼ strength potato dextrose agar (PDA) which was acidified using 1 mM lactic acid. Plated kernels were incubated in the dark for 3 days at 31 °C. Fungal colonies from the plated kernels were sub-cultured in PDA to obtain pure cultures. Only *Aspergillus* species were selected for further analysis using morphological characteristics, e.g., appearance of aerial mycelia, reverse surface, colony color and growth rate). The cultural characteristics described in taxonomic keys by Klich [48] were used to identify *Aspergillus* section *Flavi*. The isolates were sub-cultured and transferred to Aspergillus Flavus Parasiticus Agar (AFPA) as described by Muthomi, et al., [4] then incubated in the dark at 28 °C for 72 h to identify putative *Aspergillus flavus* colonies based on orange reverse color in the media.

### 5.4. DNA Extraction

The putative *Aspergillus flavus* isolates were sub-cultured in Malt Extract Agar (MEA) medium at 25 °C for three days. Mycelia were harvested from the 3-day old cultures into 1.2-mL microtubes containing six sterile beads. Microtubes containing the mycelia were capped and stored at −80 °C overnight prior to lyophilization (Christ Martin Alpha 2-4 LSCplus Lyophilizer). Microtubes with lyophilized mycelia were dipped in liquid nitrogen for 60 s and then ground into a fine powder using a Geno Grinder (SPEX SamplePrep, Metuchen, NJ, USA). Total genomic DNA was extracted using MagAttract 96 DNA Plant Core Extraction Kit (QIAGEN Inc., Mississauga, ON, Canada), following the manufacturer’s protocol. DNA quality and quantity were analyzed using 0.8% agarose gel electrophoresis and Nanodrop 2000c (Thermo Scientific, Wilmington, DE, USA) Spectrophotometry, respectively.

### 5.5. PCR Amplification and Sequencing

The DNA samples of the isolates (*n* = 218) from Section 5.4 (above) were amplified and sequenced using fungal universal primer sets of ITS1F and ITS4. A subset of 72 isolates which represented the genetic clusters of the entire collection (based on ITS sequence data) and the geographical regions were sequenced for partial calmodulin (CaM) gene (Table 3). A 50 μL volume of a reaction mixture containing AccuPower^®^ Taq PCR MasterMix (MgCl_2_-free reaction buffer, 1.5 mM MgCl_2_, 1 U of Taq polymerase, 250 μM of each dNTP), 0.2 μM of each primer and template DNA at a final concentration of 1 ng/μL was prepared. PCR was carried out as follows—first step of denaturation at 94 °C for 4 min, 35 cycles of the following three steps, 45 s at 94 °C, 45 s at specific annealing temperature (49.4 °C for ITS and 56 °C for CaM), 45 s at 72 °C, and one final extension step of 10 min at 72 °C. The PCR amplicons were assessed on 1.5% agarose gel at 100 V for 45min. PCR products were purified using QIAquick PCR Purification Kit (QIAGEN Inc., Mississauga, ON, Canada). Purified PCR products were sequenced at Bioneer Corporation Republic of South Korea. The ITS and partial calmodulin sequences were deposited to NCBI database with the accession numbers details presented in the respective results section.

### 5.6. Screening of Aflatoxin Genes

The subset of the putative *A. flavus* isolates (*n* = 72) were screened for the seven aflatoxin genes; *aflD*, *aflM*, *aflO aflP*, *aflQ*, *aflR* and *aflS* by PCR analysis using the primers in Table 3. These genes were selected with a focus of expanding the scope of the analysis of previous work. Five of the genes had been screened in previous collections, and the additional two are part of the major gene clusters [1]. The *aflD* and *aflQ* genes were amplified using two different sets of primers to test consistency of the results (Table 3). A 20 μL volume reaction mixture containing MgCl_2_-free reaction buffer, 1.5 mM MgCl_2_, 1 U of Taq polymerase, 250 μM of each dNTP, 0.2 μM of each primer and template DNA at a final concentration of 1 ng/μL was prepared. PCR was performed using the following steps: initial denaturation at 94 °C for 3 min; 30 cycles of the following three steps: denaturation at 94 °C for 1 min, annealing at 57 °C for 1 min and extension for 1 min at 72 °C; and one final extension step at 72 °C for 10 min. The PCR amplicons were assessed on 1.5% agarose gel electrophoresis at 100 V for 30 min and were viewed under UV light. The presence of an amplified band was considered to indicate the presence of the gene.

### 5.7. Aflatoxin Quantification Analysis

#### 5.7.1. Aflatoxigenicity Assay and Extraction of Aflatoxin from Culture Media

An assay was set up to quantify the amount of aflatoxin production by individual isolates on Yeast Extract Sucrose Agar (YESA) medium. Twenty-five milliliters of autoclaved YESA was dispensed in 9-cm-diameter Petri plates and allowed to cool. A loopful mycelia of *A. flavus* isolates scraped from 9-mm PDA plugs were sub-cultured on YESA plates and incubated in the dark at 28 °C for 7 days [1] to induce aflatoxin production as per the conditions established by Davis et al., [52]. Five grams of fungal plug from each sample were transferred to a sterile 50-mL Falcon tube and cut into small pieces using a sterile blade. Twenty-five milliliters of 70% methanol was added, and the slurry was shaken in a mechanical shaker at 350 rpm for 1 h. The methanolic extract in the 50-mL falcon tube was centrifuged at 3500 rpm for 10 min. The extract supernatant of volume 0.5 mL was transferred into a 2-mL centrifuge tube and diluted with 0.5 mL of 1% acetic acid prior to centrifugation at 13,000 rpm for 5 min. A total of 0.7 mL of the diluent was then transferred into HPLC vial for analysis using Ultra High-Performance Liquid Chromatography (UPLC) (Shimadzu Corporation, Kyoto, Japan).

#### 5.7.2. Extraction of Aflatoxin in Maize

To assess the level of contamination in maize samples from which *Aspergillus* had been isolated, a subset of the maize was analyzed for aflatoxin contamination. Maize samples were milled to approximately 0.5-mm particle size using a Romer Series II mill (Romer labs, Getzersdorf, Austria). Twenty-five milliliters of 70% methanol was added into the 50-mL Falcon tube (BD, Franklin Lakes, NJ, USA) containing 5 g of milled maize. The mixture was vortexed for 1 min and shaken in a mechanical orbital shaker (New Brunswick, NJ, USA) at 350 rpm for one hour at room temperature. The extract was further centrifuged at 3500 rpm for 10 min. A total of 0.5 mL of the supernatant was transferred using a micropipette into clean 2-mL centrifuge tube, diluted further with 0.5 mL of 1% acetic acid and centrifuged at 13,000 rpm for 5 min. A volume of 0.7 mL of the diluent was transferred into HPLC vial for UPLC analysis.

#### 5.7.3. Analysis Using Ultra High-Performance Liquid Chromatography 

The level of aflatoxins in naturally-contaminated maize and in the culture medium (YESA) were quantified using Ultra High-Performance Liquid Chromatography with Florescence Detection (UHPLC-FD) method. Chromatographic separation was performed using Nexera UHPLC system (Shimadzu Corporation, Kyoto, Japan) fitted with a SIL-30AC Auto sampler, LC-20AD Prominence pumps and RF-20AXS Prominence Fluorescence detector. A Synergi Hydro-RP analytical column (2.5 µm particle size, 100 mm × 3.00 mm), (Phenomenex, Torrance, CA, USA) operating at flow rate of 0.4 mL/min was used for the separation of aflatoxins. A binary mobile phase, consisting of mobile phase A methanol (40%) and Mobile phase B 1% acetic acid (60%), was utilized to achieve this separation. The injection volume was 10 μL and the column oven temperature was set at 50 °C. The liquid chromatography program was set at 8 min per run and 60% methanol was used as the flushing solution of the column. Fluorescence detection was carried out at wavelengths of λ_ex_ = 365 nm and λ_em_ = 435 nm. A standard calibration curve from a plot of peak areas against the known concentration of the injected series of standards was established and used for estimation of the concentrations of the samples in The LabSolutions software version 5.89 (Shimadzu Corporation, Kyoto, Japan, 2014). Individual types of aflatoxin were identified by comparing the retention time of the chromatographic peak of the target aflatoxin in the test sample and that of the corresponding standard chromatographic peak. Samples with values above the linear range of the standard curve were diluted and retested.

The concentration of individual aflatoxins in the test samples was calculated as follows:

(1) $$X\;\left(\mathrm{ng}/g\right)=\frac{c\;x\;V\;x\;f\;}{W}$$

where, *X*—The content of aflatoxin in the test sample in ng/g; *c*—The concentration of aflatoxin in the test sample in ng/mL; *V*—Constant volume in mL; *f*—Dilution factor of the test solution; *W*—The mass of the test sample in g.

Total aflatoxin was estimated as the sum of the concentrations of individual aflatoxin types in each sample. For quality control, certified reference maize samples and blank solvents (1:1 Mixture of 70% Methanol and 1% Acetic Acid) were included in the analysis procedure. Reference maize samples were obtained from Office of the Texas State Chemist Aflatoxin Proficiency Testing in Eastern and Central Africa program (APTECA), Chiromo Campus, Nairobi, Kenya. The accuracy of the reported aflatoxin was acceptable if the determined values of the reference materials were within the 3 ± SD in the control chart of each analytical run.

### 5.8. Data Analysis

The sequences were trimmed, assembled and edited using CLC Main Workbench 8.0.3 (https://www.qiagenbioinformatics.com). Sequences were aligned and curated using MEGA 7: Molecular Evolutionary Genetics Analysis version 7.0 for bigger datasets [53]. Redundant sequences were removed using Jalview [54]. The identity of individual isolates was inferred from comparison with the sequence database in a publicly available database (http://www.ncbi.nlm.nih.gov/). An isolate was given a species name of organism on top hit at a sequence homology of greater than 99%. Phylogenetic trees based on Maximum Likelihood were constructed based on the best model using both MEGA 7 and R software package. Using R software, the trimmed, assembled, aligned and edited sequences of ITS and CaM were merged. Phylogenetic analyses were performed on these data to obtain genetic diversity among the isolates and the number of unique alleles/sequences per region. The data were also related to the toxigenic potential of the isolates, CaM sequences and ITS sequences. Linear regression was used to test the correlation between the actual aflatoxin contamination of the maize and the toxigenicity of the *Aspergillus* species recovered from the same maize sample. A stepwise linear regression model was used to determine the effect of individual aflatoxin genes on the toxigenicity of the isolates. AIC regression was first applied to select the best model by estimating the quality of each model, relative to each of the other models.

## Figures and Tables

**Table 1:** Positions of the partial calmodulin gene showing single nucleotide polymorphisms (SNPs) that can discriminate between *Aspergillus minisclerotigenes* and *Aspergillus flavus* isolates from three regions of Kenya.

Nucleotide Position in Calmodulin Sequence	39	144	424	466	691	701
***A. minisclerotigenes***	A(3)/G(6)	G	G	G(7)/C(2)	A(4)/G(5)	A
***A. flavus***	G	A(60)/G(3)	A	C	A	T

**Table 2:** Association between the aflatoxin biosynthetic genes and the aflatoxin production potential in Kenyan *Aspergillus flavus* and *Aspergillus minisclerotigenes* isolates. Gene effects were compared in a stepwise regression model and the best model was selected based on the Akaike information criterion [31].

Gene Effect	Magnitude of Gene Effect	Std Error of the Effect	*t*-Value	Significance *p*-Value
*aflD*’	−0.8736	0.4439	−1.968	0.053
*aflD*”	3.4093	1.162	2.934	0.005
*aflM*	−0.239	0.6628	−0.361	0.720
*aflR*	0.4392	0.7546	0.582	0.563
*aflS*	2.4061	0.7943	3.029	0.004
*aflD*” and *aflM*	−2.109	1.4374	−1.467	0.147
*aflD*” and *aflR*	−2.2789	1.0133	−2.249	0.028
Intercept	1.4965	0.423	3.538	0.001

**Table 3:** DNA sequences of the primers used in the study.

Target Gene	Primer Code	Primer DNA Sequence	Size (bp)	Reference
*ITS*	ITS 1F	5′-CTTGGTCATTTAGAGGAAGTAA-3′	595	[49]
	ITS4	5′-TCCTCCGCTTATTGATATGC-3′		
*CaM*	CF1M	5′-AGGCCGAYTCTYTGACYGA-3′	700	[50]
	CF4	5′-TTTYTGCATCATRAGYTGGAC-3′		
*AflD*	AflD-1	5′-CACTTAGCCATCACGGTCA-3′	852	[27]
	AflD-2rev	5′-GAGTTGAGATCCATCCGTG-3′		
	Nor1-F	5′-ACCGCTACGCCGGCACTCTCGGCAC-3′	400	[15]
	Nor1-R	5′-GTTGGCCGCCAGCTTCGACACTCCG -3′		
*AflM*	AflM-1	5′-AAGTTAATGGCGGAGACG-3′	470	[27]
	AflM-2rev	5′-TCTACCTGCTCATCGGTGA-3′		
*AflO*	AflO-1	5′-TCCAGAACAGACGATGTGG-3′	790	[27]
	AflO-2rev	5′-CGTTGGCTAGAGTTTGAGG-3′		
*AflP*	AflP-1	5′-AGCCCCGAAGACCATAAAC-3′	870	[27]
	AflP-2rev	5′-CCGAATGTCATGCTCCATC-3′		
*AflQ*	AflQ-1	5′-TCGTCCTTCCATCCTCTTG-3′	757	[27]
	AflQ-2rev	5′-ATGTGAGTAGCATCGGCATTC-3′		
	Ord1-gF	5′-TTA AGG CAG CGG AAT ACA AG-3′	719	[51]
	Ord1-gR	5′-GAC GCC CAA AGC CGA ACA CAA A-3′		
*AflR*	AflR-1	5′-AAGCTCCGGGATAGCTGTA-3′	1079	[27]
	AflR-2rev	5′-AGGCCACTAAACCCGAGTA-3′		
*AflS*	AflS-1	5′-TGAATCCGTACCCTTTGAGG-3′	684	[27]
	AflS-2rev	5′-GGAATGGGATGGAGATGAGA-3′		

## Supplementary Materials

The following are available online at https://www.mdpi.com/2072-6651/11/8/467/s1, Table S1: Clusters of *Aspergillus flavus* isolates based on ITS sequences showing all the isolates in each cluster, Table S2: Clusters of *Aspergillus flavus* isolates with the positions showing nucleotide substitutions, deletions and insertions on ITS gene region, Table S3: *Aspergillus flavus* isolates with positions showing nucleotide substitutions, deletions and insertions on calmodulin gene region, Figure S1: Aflatoxin types produced by high aflatoxigenic *Aspergillus flavus* isolates, Figure S2: Aflatoxin types produced by low aflatoxigenic *Aspergillus flavus* isolates, Figure S3: A boxplot showing the comparison of the total Aflatoxin production potential between *Aspergillus flavus* and *Aspergillus minisclerotigenes*, Figure S4: A graph of distinct alleles of *Aspergillus flavus* isolates observed in each of three region, and Figure S5: Map of Kenya showing the sampling sites (red dots) within the three geographical regions.
