# Supplementary material for: Genetic Profiling of Aspergillus Isolates with Varying Aflatoxin Production Potential from Different Maize-Growing Regions of Kenya
Source: Toxins (Basel). 2019 Aug 9;11(8):467. doi: 10.3390/toxins11080467 (PMC6723045; doi:10.3390/toxins11080467)
Supplement: Supplementary file 1 [file toxins-11-00467-s001.pdf]

Supplementary Materials: Genetic Profiling of *Aspergillus* Isolates with Varying Aflatoxin Production Potential from Different Maize Growing Regions of Kenya

Richard Dooso Oloo, Sheila Okoth, Peter Wachira, Samuel Mutiga, Phillis Ochieng, Leah Kago, Fredrick Nganga, Jean-Baka Domelevo Entfellner and Sita Ghimire

Table S1. Clusters of *Aspergillus flavus* isolates based on ITS sequences showing all the isolates in each cluster.

Cluster	Isolate Code	Total Isolates	NCBI Identity
**1**	26	1	*A. flavus*
**4**	42	1	*A. flavus*
**2**	167	1	*A. flavus*
**3**	199	1	*A. flavus*
**5**	16, 160, 181, 21, 216, 219, 22, 224, 225, 25, 36, 39, 52, 64	14	*A. flavus*
**6**	161	1	*A. flavus*
**7**	104, 105, 106, 118, 120, 4, 5, 60, 62, 63, 72	11	*A. flavus*
**8**	1, 10, 100, 101, 102, 103, 107, 108, 109, 11, 110, 111, 112, 113, 114, 115, 116, 117, 119, 12, 121, 122, 123, 124, 125, 126, 127, 128, 129, 13, 130, 131, 132, 133, 134, 137, 138, 139, 14, 140, 141, 142, 145, 146, 147, 148, 149, 15, 150, 151, 152, 153, 154, 155, 156, 157, 158, 159, 162, 163, 164, 165, 168, 17, 170, 171, 172, 173, 174, 179, 18, 182, 183, 184, 185, 186, 187, 188, 189, 19, 190, 191, 192, 193, 194, 195, 196, 197, 198, 2, 20, 200, 201, 202, 203, 204, 205, 207, 208, 209, 210, 211, 212, 213, 214, 215, 217, 218, 220, 221, 222, 223, 226, 227, 228, 229, 23, 230, 231, 232, 233, 234, 235, 24, 28, 29, 3, 30, 31, 32, 33, 34, 35, 37, 38, 40, 41, 43, 44, 45, 46, 47, 48, 49, 50, 51, 53, 54, 55, 56, 57, 58, 59, 6, 61, 65, 66, 67, 68, 69, 7, 70, 71, 73, 74, 75, 76, 77, 78, 79, 8, 85, 86, 87, 88, 89, 9, 90, 91, 92, 93, 95, 96, 97, 98, 99	186	*A. flavus*
**9**	206	1	*A. flavus*
**10**	135	1	*A. flavus*

Table S2. Clusters of *Aspergillus flavus* isolates with the positions showing nucleotide substitutions, deletions and insertions on ITS gene region

Isolate Cluster Group	Position and type of SNP observed	Total No. of SNPs
Cluster 1	77: Substitution of C with A	1
Cluster 2	77: Substitution of C with A<br>87: Substitution of G with C	2
Cluster 3	38: Substitution of A with C<br>48: Insertion of A	2
Cluster 4	490: Substitution of C with A	1
Cluster 6	63: Substitution of C with T	2

	466: Substitution of T with C	
Cluster 7	29: Substitution of C with T<br>399: Substitution of T with C	2
Cluster 8	466: Substitution of T with C	1
Cluster 9	122, 164, 175, 391, 406, 450 and 587: Substitution of G with A<br>133: Substitution of G with C	8
Cluster 10	164, 172, 175, 266, 274, 344, 346, 376, 391, 401, 406, 410, 483, and 587: Substitution of G with A<br>189, 333, 452 and 486: Substitution of G with C	18

Table S3. *Aspergillus flavus* isolates with positions showing nucleotide substitutions, deletions and insertions on Calmodulin gene region

NCBI identity	Isolates	Nucleotide Position and Polymorphism Type	Total No. of SNPs
A. flavus	8, 76, 74	105, 256 and 532: Substitution of G with A<br>144: Substitution of A with G	4
A. flavus	73, 4, 44, 52, 6, 60, 62, 72, 225, 223, 22, 218, 216, 206, 199, 185, 179, 161, 160, 156, 152, 147,137, 128, 120, 102, 104	222 and 533: Substitution of G with A<br>543: Substitution of A with T<br>557: Insertion of A	4
A. flavus	181, 107	16: Substitution of C with G<br>447: Substitution of G with A	2
A. flavus	165, 157	30, 33, 222 and 530: Substitution of G with A<br>34: Insertion of G<br>35: Substitution of C with T<br>541: Substitution of A with T<br>557: Insertion of A	8
A. flavus	42, 141, 167, 207, 36, 39	16: Substitution of C with G	1
A. flavus	135	222 and 531: Substitution of G with A<br>542: Substitution of A with T<br>557: Insertion of A<br>576: Substitution of A with C	5
A. flavus	56	508: Substitution of T with A<br>607: Insertion of A	2
A. minisclerotigenes	145	2: Substitution of A with C<br>39, 255, 303: Substitution of G with A<br>144 and 424: Substitution of A with G<br>701: Substitution of T with A<br>672: Substitution of T with C<br>15: Substitution of C with T	9
A. minisclerotigenes	25	2: Substitution of A with C<br>39, 205 and 303: Substitution of G with A<br>144 and 424: Substitution of A with G<br>701: Substitution of T with A	7
	231	39 and 255: Substitution of G with A<br>144 and 424: Substitution of A with G	11

A. minisclerotigenes		701: Substitution of T with A<br>203 and 218: Substitution of C with T<br>243 and 466: Substitution of C with G<br>344 and 437: Substitution of G with T	
A. minisclerotigenes	138, 64	144 and 424: Substitution of A with G<br>255: Substitution of G with A<br>701: Substitution of T with A<br>466: Substitution of C with G	5
A. minisclerotigenes	88, 86, 85, 78	144 and 424: Substitution of A with G<br>255 and 691: Substitution of G with A<br>701: Substitution of T with A<br>466: Substitution of C with G	6

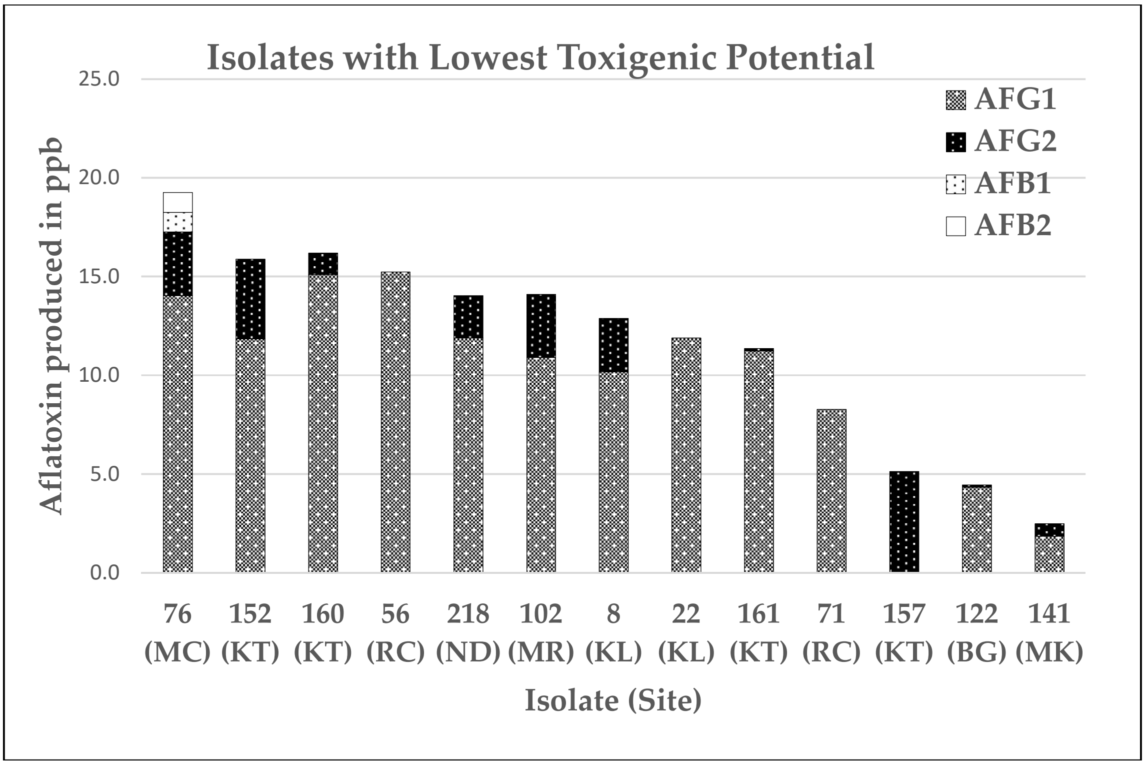

Figure S1. Aflatoxin types produced by low aflatoxigenic *Aspergillus flavus* isolates. RC=Rachuonyo, BG=Bungoma, KT=Kitale, ND=Nandi, MC Machakos, MK=Makueni, MR=Meru) and KL=Kilifi.

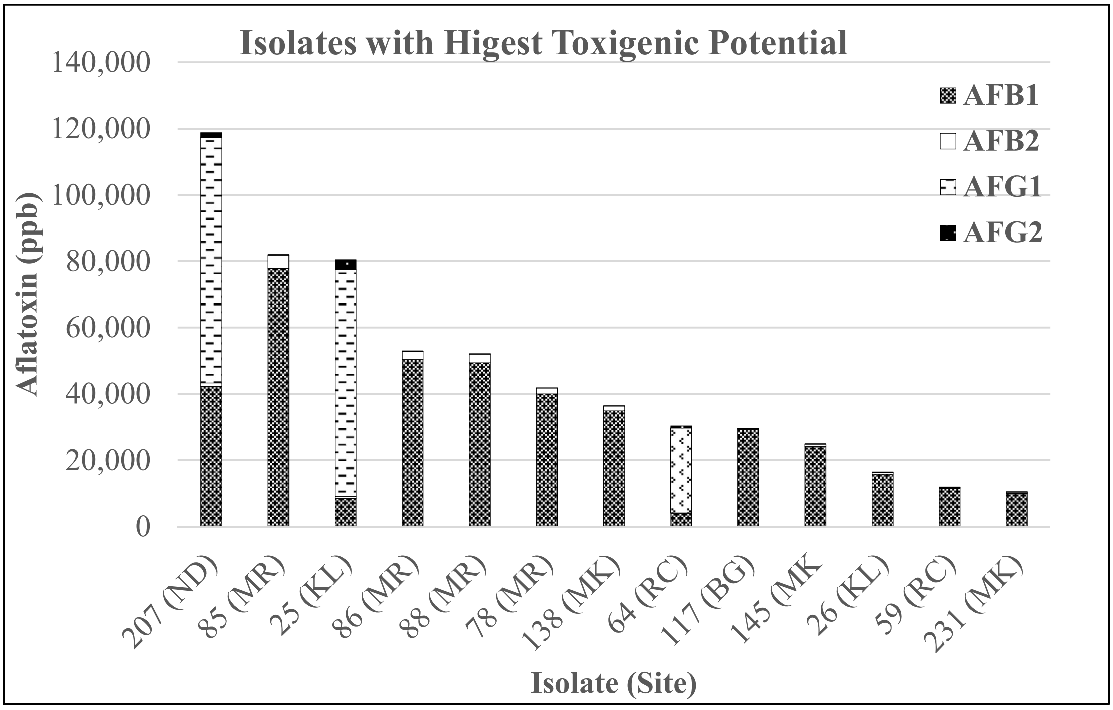

Figure S2. Aflatoxin types produced by high aflatoxigenic *Aspergillus spp.* isolates. RC=Rachuonyo, BG=Bungoma, KT=Kitale, ND=Nandi, MC Machakos, MK=Makueni, MR=Meru) and KL=Kilifi.

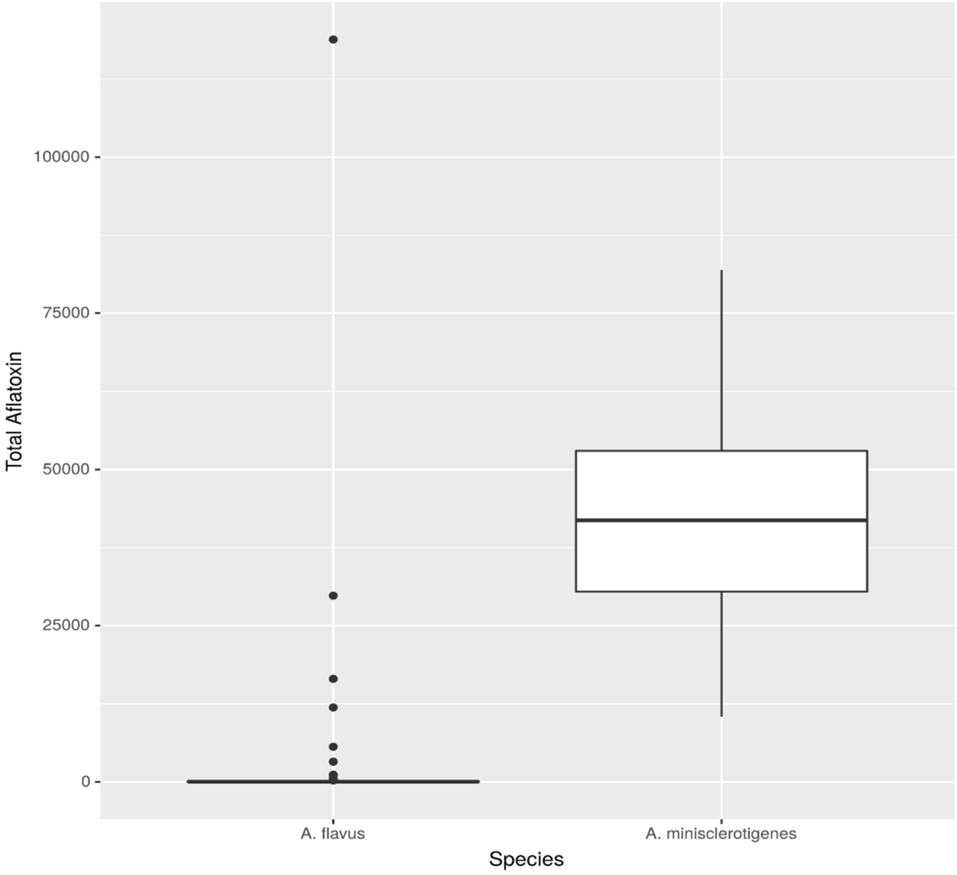

Figure S3. A boxplot showing the comparison of the total Aflatoxin production potential between *Aspergillus flavus* and *Aspergillus minisclerotigenes*

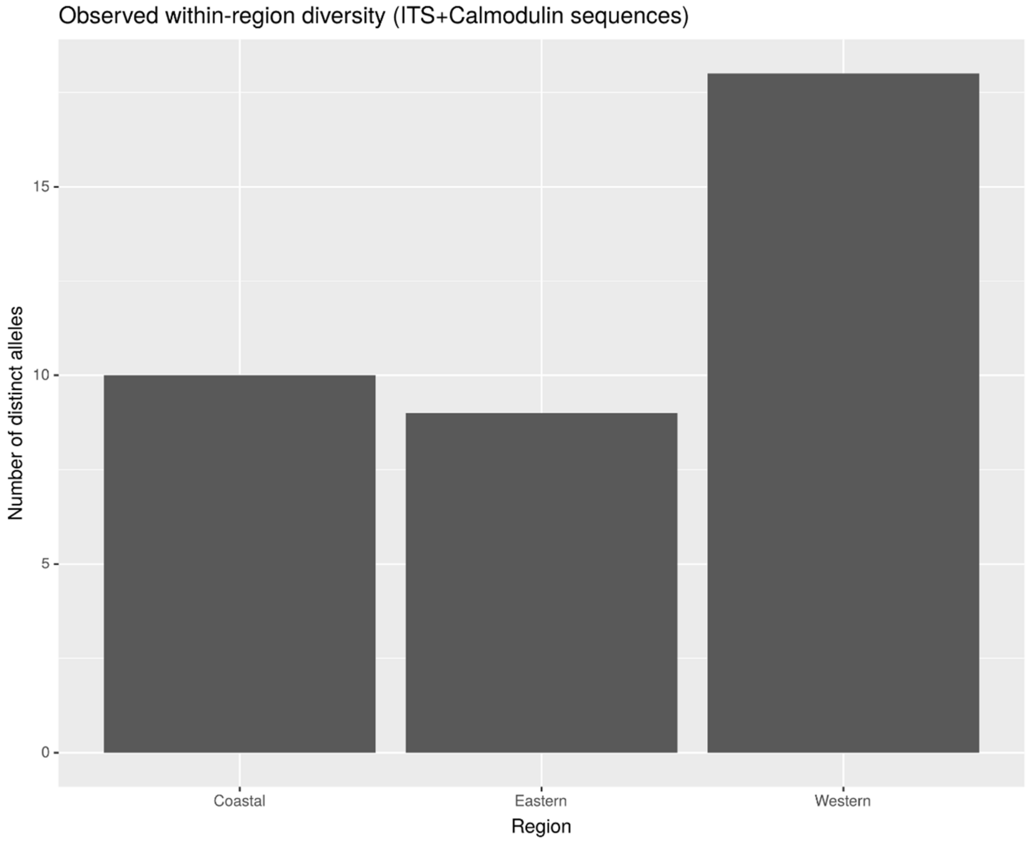

Figure S4. A graph of distinct alleles of *Aspergillus flavus* isolates observed in each of three region.

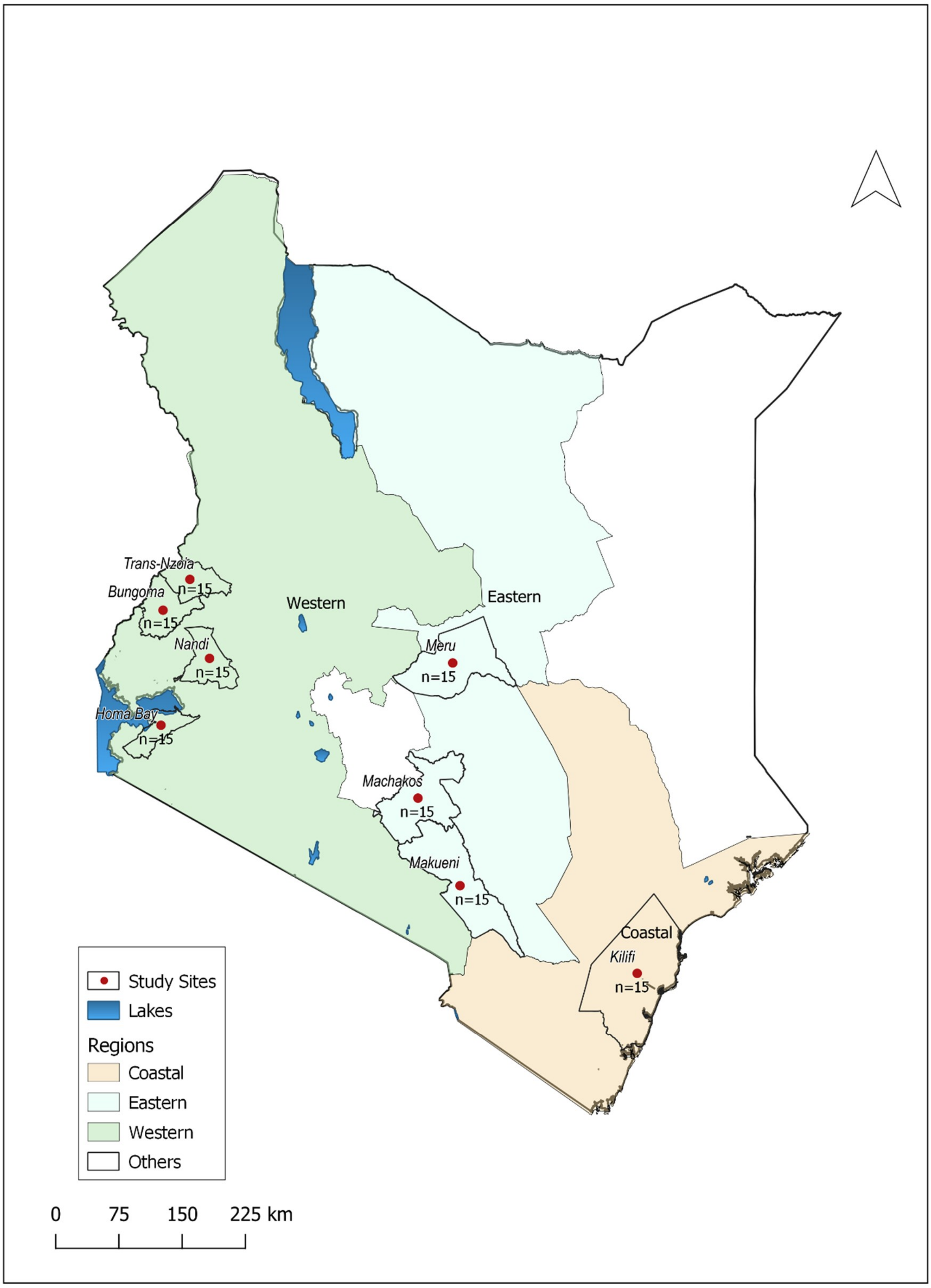

Figure S5. Map of Kenya showing the sampling sites (red dots) within the three geographical regions.
